# Bioprosthetic valve replacement for concomitant aortic and mitral positions

**DOI:** 10.1016/j.xjon.2025.04.013

**Published:** 2025-04-23

**Authors:** Hong Rae Kim, Ha Eun Oh, Ho Jin Kim, Seon-Ok Kim, Ye-Jee Kim, Jung-Min Ahn, Joon Bum Kim, Dae-Hee Kim

**Affiliations:** a Department of Thoracic and Cardiovascular Surgery, Asan Medical Center, University of Ulsan College of Medicine, Seoul, Republic of Korea; b Division of Cardiology, Asan Medical Center, University of Ulsan College of Medicine, Seoul, Republic of Korea; c Department of Clinical Epidemiology and Biostatistics, Asan Medical Center, University of Ulsan College of Medicine, Seoul, Republic of Korea

**Keywords:** heart valve prosthesis, aortic valve replacement, mitral valve replacement

## Abstract

**Objective:**

To compare the clinical outcomes of double-valve replacement (DVR) using bovine pericardial and porcine bioprostheses, using a nationwide administrative claims database.

**Methods:**

Adult patients (age ≥40 years) who underwent bioprosthetic DVR between 2003 and 2018 were identified from the Korean National Health Insurance Service database. The outcomes of interest were all-cause mortality, cardiac mortality, and valve-related events, including the incidences of reoperation, endocarditis, systemic thromboembolism, and major bleeding. Baseline adjustment was performed using propensity score matching. Time-related outcomes were evaluated using a competing risk analysis, with death considered a competing risk.

**Results:**

Among the 889 patients who met the inclusion criteria, 608 (68.3%) received a bovine pericardial valve and the other 281 (31.6%) received a porcine valve. After matching 195 pairs of patients, there were no significant differences in cardiovascular mortality, all-cause mortality, thromboembolism, or major bleeding between the bovine and porcine groups; however, patients with porcine valves had a higher risk of reoperation (adjusted hazard ratio, 2.08; 95% confidence interval, 1.10-3.94) in competing risk analyses. An adjusted subgroup analysis showed that patients without diabetes and a lower Charlson Comorbidity Index who received a porcine valve had a higher risk of reoperation.

**Conclusions:**

This nationwide cohort study on DVR revealed that the choice of bioprosthetic valve type was not associated with the risk of cardiovascular mortality. However, the use of porcine prostheses was significantly associated with a higher risk of reoperation.

**Figure:**
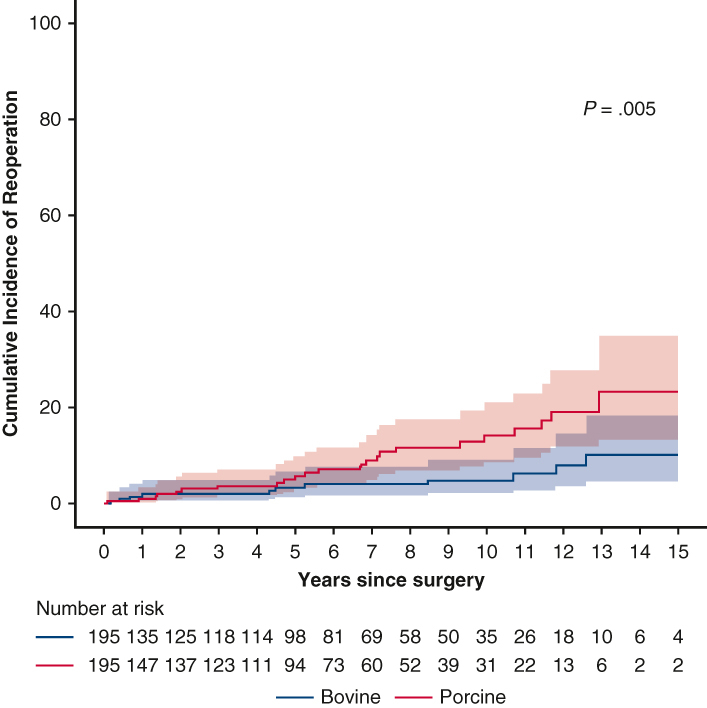
Cumulative reoperation incidence in matched cohorts. The shaded area represents 95% confidence interval.

**Central Message**

Using porcine prostheses may be associated with an increased risk of reoperation compared to bovine prostheses in double-valve replacement.

**Perspective**

The use of bioprosthetic valves in double-valve replacement (DVR) may yield different outcomes compared to isolated aortic valve replacement or mitral valve replacement performed separately. In this nationwide cohort study on DVR, we found that bioprosthetic valve type was not associated with the risk of cardiovascular mortality; however, the use of porcine prostheses was significantly associated with a higher risk of reoperation.

Multivalvular heart disease, affecting both the aortic and mitral valves, carries a significant morbidity and mortality burden in individuals with valvular heart disease, accounting for approximately 17% of patients treated for valvular disease.^1^^,^^2^ In elderly patients undergoing heart valve replacement, bioprosthetic valves are preferred because they do not require lifelong anticoagulation. Given the demographic characteristics of multivalve diseases, which afflict predominately elderly patients, there is a growing trend toward the use of bioprosthetic valves in these cases.

The 2 most commonly used types of biological valves are porcine xenograft valves and bovine pericardial tissue valves. There currently are no specific recommendations based on valve material, however. Moreover, prior studies that offer guidance on valve selection, particularly regarding valve material, have focused primarily on single-valve scenarios, thereby leaving the decision making process for multiple valve diseases largely dependent on surgeon preference.^3^^,^^4^

To address this knowledge gap, we conducted a comprehensive nationwide cohort study using the national administrative database of the Korean National Health Insurance Service (NHIS) to compare the long-term clinical outcomes of these 2 valve types in double-valve replacement (DVR).

## Patients and Methods

### Data Sources

The study data were obtained from the Korean National Health Information Database (NHID), which is maintained by the NHIS. The NHIS is the sole institution that provides coverage to almost 100% of the national population in South Korea, which amounted to approximately 52 million people in 2022.^5^ Therefore, using the NHID ensures extensive and long-term follow-up data while minimizing participation bias. The NHID encompasses health-related information including demographic information, diagnoses, treatments, prescriptions, and health screening records.

In Korea, adults age ≥19 are required to undergo regular biannual health screenings. These examinations include such measurements as height, weight, body mass index, blood pressure, and pulse rate. Laboratory tests include a complete blood count, serum glucose, serum cholesterol/triglyceride levels, serum creatinine, and liver function tests. Health screenings typically include electrocardiography, chest radiography, and self-reported questionnaires on health behaviors, such as smoking and alcohol use. All diagnoses are recorded in the form of International Classification of Diseases, Tenth Revision (ICD-10) codes.

The study was approved by the Institutional Review Board of Asan Medical Center (approval 2024-0420; approved March 26, 2024). Given that the NHID provides anonymized datasets in which personal information cannot be discerned, the requirement for informed consent was waived.

### Patients

**Figure 1:**
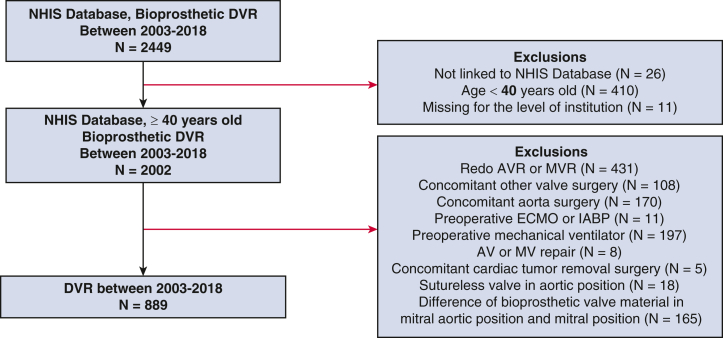
Flow diagram of the study patients. *NHIS*, National Health Insurance Service; *DVR*, double-valve replacement; *AVR*, aortic valve replacement; *MVR*, mitral valve replacement; *ECMO*, extracorporeal membrane oxygenation; *IABP*, intra-aortic balloon pump; *AV*, aortic valve; *MV*, mitral valve.

The study cohort comprised adults age ≥40 years who underwent DVR (aortic and mitral) with a bioprosthetic valve between January 2003 and December 2018. The exclusion criteria were (1) redo aortic valve replacement (AVR) or redo mitral valve replacement (MVR), (2) concomitant other valve surgery other than tricuspid valve repair, (3) concomitant aorta surgery, (4) use of preoperative extracorporeal membrane oxygenation or intra-aortic balloon pump, (5) preoperative mechanical ventilator, (6) aortic valve or mitral valve repair, (7) concomitant cardiac tumor removal surgery, (8) AVR with a sutureless valve, and (9) difference of bioprosthetic valve material in mitral aortic position and mitral position (Figure 1). Further details on the types of valves used in the study are provided in Table E1.

### Study Endpoints

Study endpoints included death, cardiac death, and valve-related events, such as endocarditis, reoperation, thromboembolism, and major bleeding. Data on deaths and their causes were obtained from Statistics Korea, which maintains records of residents in South Korea using personal identification numbers. Cardiovascular death was defined as a circulatory system disease without exceptions, according to the ICD-10 (ICD code I10-99). Reoperation was defined as a surgical reintervention involving either the mitral valve or aortic valve during the follow-up period. Notably, the valve-in-valve procedure was not available in Korea during the study period. Therefore, all reoperations in this study refer exclusively to surgical reinterventions. Thromboembolism events included both ischemic stroke and systemic thromboembolism (ICD codes I63, I64, and I74). Ischemic stroke was confirmed using ICD-10 codes (I63-64) and NHIS claim codes, along with corroborative brain imaging (computed tomography or magnetic resonance imaging) during hospitalization. Systemic thromboembolism was defined as arterial embolism and thrombosis (ICD code I74) and confirmed through imaging during hospitalization. Major bleeding included image-confirmed brain hemorrhage, gastrointestinal bleeding, and bleeding at other sites requiring hospitalization. Tables E2 and E3 provide definitions of variables and outcomes based on ICD-10 and NHIS codes.

### Statistical Analysis

Categorical variables are presented as frequency and percentage. Comparisons were conducted using either the χ^2^ test or Fisher exact test, depending on sample size and data distribution. The normality of continuous variables was assessed using the Shapiro-Wilk test. Variables with a normal distribution were compared using the Student *t* test and are expressed as mean ± standard deviation. Missing data from health screenings was considered a categorical variable and analyzed accordingly.

Propensity score (PS) matching was used to minimize bias caused by confounding variables and to balance baseline characteristics between the bovine pericardial and porcine groups. PSs were estimated using a logistic regression model and applied to incorporate the baseline characteristics listed in Table 1. To address the issue of missing health screening data for certain patients, we consolidated the unavailable data into a unified categorical variable labeled “not available.” This strategy was used to maintain a balanced distribution of missing data across both groups during PS matching. Patients were matched using the nearest-neighbor method without replacement, using a caliper width set at 0.2 times the pooled standard deviation of the logit of the PS. A standardized mean difference <0.1 was considered indicative of well-balanced baseline variables between the 2 bioprosthetic valve groups.

**Table 1:** Baseline characteristics according to bioprosthetic valve type

Variable	Unadjusted data				Propensity score matching		
	**Bovine group** **(N = 608)**	**Porcine group** **(N = 281)**	***P* value**	**SMD**	**Bovine group** **(N = 195)**	**Porcine group** **(N = 195)**	**SMD**
Baseline demographics							
Age, y, mean ± SD	69.56 ± 7.83	70.64 ± 5.93	.029	0.150	70.30 ± 6.81	70.36 ± 6.28	0.009
Female sex, n (%)	365 (60.0)	161 (27.3)	.110	0.056	114 (58.5)	115 (59.0)	0.01
Baseline comorbidities, n (%)							
Hypertension	395 (65.0)	196 (69.8)	.160	0.102	134 (38.7)	133 (68.2)	0.011
Diabetes mellitus	138 (22.7)	65 (23.1)	.886	0.010	39 (20.0)	42 (21.5)	0.038
Dyslipidemia	103 (16.9)	49 (17.4)	.855	0.013	37 (19.0)	35 (17.9)	0.026
Atrial fibrillation	246 (40.5)	112 (39.9)	.865	0.012	83 (42.6)	78 (40.0)	0.052
Chronic kidney disease	45 (7.4)	8 (2.8)	.008	0.208	8 (4.1)	6 (3.1)	0.055
Dialysis	27 (4.4)	6 (2.1)	.091	0.130	7 (3.6)	4 (2.1)	0.093
Ischemic stroke	100 (16.4)	45 (16.0)	.871	0.012	32 (16.4)	31 (15.9)	0.014
Ischemic heart disease	201 (33.1)	96 (34.2)	.745	0.023	66 (33.8)	65 (33.3)	0.011
Myocardial infarction	19 (3.1)	12 (4.3)	.387	0.061	8 (4.1)	7 (3.6)	0.027
Previous PCI	25 (4.1)	13 (4.6)	.724	0.025	8 (4.1)	11 (5.6)	0.072
Congestive heart failure	302 (49.7)	132 (47.0)	.455	0.054	89 (45.6)	97 (49.7)	0.082
Anemia	89 (14.6)	31 (11.0)	.143	0.108	22 (11.3)	22 (11.3)	<0.001
COPD	38 (6.3)	27 (9.6)	.074	0.125	16 (8.2)	14 (7.2)	0.038
Asthma	137 (22.5)	59 (21.0)	.607	0.037	41 (21.0)	41 (21.0)	<0.001
Peripheral vascular disease	45 (7.4)	23 (8.2)	.683	0.029	14 (7.2)	13 (6.7)	0.02
Previous cancer	43 (7.1)	15 (5.3)	.330	0.072	15 (7.7)	14 (7.2)	0.059
CCI, n (%)			.753	0.099			0.103
0	100 (16.4)	44 (15.7)			35 (17.9)	33 (16.9)	
1	137 (22.5)	62 (22.1)			42 (21.5)	46 (23.6)	
2	119 (19.6)	66 (23.5)			47 (24.1)	40 (20.5)	
3-4	163 (26.8)	72 (25.6)			49 (25.1)	51 (26.2)	
≥5	89 (14.6)	37 (13.2)			22 (11.3)	25 (12.5)	
Mode of valve disease, n (%)							
Aortic stenosis	289 (47.5)	121 (43.1)	.214	0.090	93 (47.7)	84 (43.1)	0.093
Aortic regurgitation	267 (43.9)	116 (41.3)	.461	0.053	83 (42.6)	82 (42.1)	0.01
Combined (aortic valve)	131 (21.5)	63 (22.4)	.769	0.021	45 (23.1)	37 (20.0)	0.075
Mitral stenosis	451 (74.2)	186 (66.2)	.014	0.175	138 (70.8)	139 (71.3)	0.011
Mitral regurgitation	57 (9.4)	40 (14.2)	.031	0.151	25 (12.8)	23 (11.8)	0.031
Combined (mitral valve)	150 (24.7)	70 (24.9)	.939	0.006	48 (24.6)	50 (25.6)	0.024
Health screening data							
BMI, kg/m^2^							
Mean ± SD	23.51 ± 3.43	22.86 ± 2.84	.024	0.206	22.75 ± 3.05	22.92 ± 2.87	0.058
<18.5, n (%)	24 (3.9)	11 (3.9)	.151	0.222	10 (5.1)	8 (4.1)	0.128
≥18.5 and <23, n (%)	137 (22.5)	71 (25.3)			47 (24.1)	50 (25.6)	
≥23 and <25, n (%)	106 (17.4)	45 (16.0)			30 (15.4)	33 (16.9)	
≥25 and <30, n (%)	90 (14.8)	31 (11.0)			24 (12.3)	22 (11.3)	
≥30, n (%)	14 (2.3)	1 (0.4)			0 (0.0)	1 (0.5)	
Not available, n (%)	237 (39.0)	122 (43.4)			84 (43.1)	81 (41.5)	
Systolic blood pressure, n (%)			.920	0.009			0.034
<120 mm Hg	125 (20.6)	57 (20.3)			41 (21.0)	42 (21.5)	
≥120 and <140 mm Hg	177 (29.1)	72 (25.6)			47 (24.1)	51 (26.2)	
≥140 mm Hg	69 (11.3)	30 (10.7)			23 (11.8)	21 (10.8)	
Not available	237 (39.0)	122 (43.4)			84 (43.1)	81 (41.5)	
Diastolic blood pressure, n (%)			.593	0.051			0.08
<80 mm Hg	232 (38.2)	99 (35.2)			62 (31.8)	69 (35.4)	
≥80 and <90 mm Hg	101 (16.6)	45 (16.0)			34 (17.4)	33 (16.9)	
≥90 mm Hg	38 (6.3)	15 (15.3)			15 (7.7)	12 (6.2)	
Not available	237 (39.0)	122 (43.4)			84 (43.1)	81 (41.5)	
Smoking status, n (%)			.601	0.100			0.036
Never smoking	276 (45.4)	125 (44.5)			84 (43.1)	86 (44.1)	
Previous smoker	50 (8.2)	17 (6.0)			15 (7.7)	15 (7.7)	
Current smoker	35 (5.8)	15 (5.3)			10 (5.1)	11 (5.6)	
Not available	247 (40.6)	124 (44.1)			86 (44.1)	83 (42.6)	
Alcohol use, n (%)			.014	0.236			0.036
None	237 (39.1)	83 (29.5)			58 (29.7)	61 (31.3)	
Mild to moderate	107 (17.6)	69 (24.6)			48 (24.6)	48 (24.6)	
Heavy	16 (2.6)	5 (1.8)			3 (1.5)	3 (1.5)	
Not available	248 (40.8)	124 (44.1)			86 (44.1)	83 (42.6)	
Creatinine concentration, n (%)			.014	0.217			0.055
≤1.5 mg/dL	266 (43.8)	101 (35.9)			68 (34.9)	72 (36.9)	
>1.5 mg/dL	17 (2.8)	3 (1.1)			4 (2.1)	3 (1.5)	
Not available	325 (53.5)	177 (63.0)			123 (63.1)	120 (61.5)	

*SMD*, Standardized mean difference; *SD*, standard deviation; *PCI*, percutaneous coronary intervention; *COPD*, chronic obstructive pulmonary disease; *CCI*, Charlson Comorbidity Index; *BMI*, body mass index.

After adjustment with PS matching, the Cox proportional hazard model with robust standard errors was used to compare the risk of all-cause mortality between the 2 groups. The Fine and Gray method was used to analyze the risk of time-related outcomes, taking into account cardiovascular mortality as a competing event. To address potential unmeasured confounders that could have influenced the impact of valve type, we performed a sensitivity analysis using falsification endpoints, including chronic obstructive pulmonary disease, pneumonia, and lower extremity fracture. This method was described in detail in our previous study.^6^^,^^7^

Subgroup analyses were conducted to compare the outcomes of the 2 bioprosthetic valve groups across various baseline risk factors. In these comparisons, separate cohorts were established using inverse probability of treatment weighting adjustment to mitigate potential selection bias, especially in subgroups with smaller sample sizes.

All *P* values were 2-tailed, and *P* < .05 was considered to indicate statistical significance. All statistical analyses were performed using R version 4.0.3 (R Foundation for Statistical Computing) and SAS Enterprise Guide version 7.1 (SAS Institute).

## Results

### Patient Characteristics

During the initial enrollment, a total of 2449 patients who underwent DVR with a bioprosthetic valve were identified. After applying our exclusion criteria, 889 patients were included in this study (Figure 1). Among these, 608 (68.3%) received a bovine pericardial valve and the other 281 (31.6%) received a porcine valve. Table 1 presents the baseline characteristics of the patients. Before matching, individuals who received a bovine pericardial valve tended to have chronic kidney disease and congestive heart failure and were more likely to be treated in larger-volume centers compared to those who received a porcine valve. Additionally, there was a trend toward increasing use of bovine prostheses over the study period. No difference in concomitant surgical procedures was observed between the 2 groups (Table 2). After matching, each group contained 195 patients, and most baseline covariates were well-balanced.

**Table 2:** Operative profile according to bioprosthetic valve type

Variable	Unadjusted data				Propensity score-matched		
	Bovine group (N = 608)	Porcine group (N = 281)	*P* value	SMD	Bovine group (N = 195)	Porcine group (N = 195)	SMD
Concomitant procedure, n (%)							
Surgical ablation	207 (34.0)	98 (34.9)	.81	0.017	67 (34.4)	67 (34.4)	<0.001
TV repair	227 (37.3)	90 (32.0)	.13	0.112	60 (30.8)	64 (32.8)	0.044
CABG	37 (6.1)	23 (8.2)	.25	0.082	15 (7.7)	12 (6.2)	0.061

*SMD*, Standardized mean difference; *TV*, tricuspid valve; *CABG*, coronary artery bypass grafting.

### Clinical Outcomes

Table 3 presents the incidence and risk analysis of clinical outcomes. Over a median follow-up of 5.74 years (interquartile range [IQR], 3.19-9.79 years), 374 patients died (7.7%/person-year [PY]), with 235 of these deaths attributed to cardiac causes (4.8%/PY). Before PS matching, the mean duration of follow-up in the 889 patients was 5.23 PY in the bovine pericardial valve group and 6.05 PY in the porcine valve group. After PS matching, the mean duration of follow-up was 5.29 PY in the bovine group and 5.15 PY in the porcine group. The total after matching was 1031.4 PY for the bovine group and 1004.2 PY for the porcine group.

**Table 3:** Clinical outcomes in the bovine and porcine groups

Outcome	Unadjusted data				Propensity score-matched			
	No. of events (%/PY)		HR (95% CI)	*P* value	No. of events (%/PY)		aHR (95% CI)	*P* value
	Bovine (N = 608)	Porcine (N = 281)			Bovine (N = 195)	Porcine (N = 195)		
Death	238 (7.5)	136 (8.0)	1.16 (0.94-1.43)	.17	100 (8.5)	87 (7.4)	0.87 (0.65-1.17)	.37
Cardiovascular death	152 (4.8)	83 (4.9)	1.10 (0.84-1.45)	.45	68 (5.8)	51 (4.3)	0.74 (0.50-1.07)	.10
Noncardiovascular death	86 (2.7)	53 (3.1)	1.15 (0.83-1.62)	.40	32 (2.7)	36 (3.1)	1.20 (0.76-1.92)	.43
Valve-related events								
Endocarditis	7 (0.3)	10 (0.7)	2.82 (1.07-7.45)	.036	3 (0.3)	7 (0.7)	2.31 (0.59-9.10)	.23
Reoperation	32 (1.2)	27 (1.8)	1.56 (0.94-2.58)	.08	12 (1.2)	23 (2.3)	2.08 (1.10-3.94)	.025
Thromboembolism	39 (1.5)	21 (1.4)	0.97 (0.57-1.65)	.91	13 (1.3)	11 (1.1)	0.88 (0.40-1.93)	.74
Hemorrhage	156 (7.2)	83 (6.7)	0.98 (0.75-1.27)	.86	56 (7.1)	59 (6.9)	1.07 (0.76-1.53)	.70
Falsification endpoint								
COPD	15 (0.5)	9 (0.6)	1.09 (0.48-2.48)	.83	4 (0.4)	3 (0.3)	0.77 (0.17-3.48)	.73
Pneumonia	75 (2.9)	56 (3.9)	1.38 (0.98-1.96)	.07	32 (3.2)	39 (4.0)	1.30 (0.82-2.07)	.27
Fracture	24 (0.9)	10 (0.7)	0.71 (0.34-1.47)	.36	12 (1.2)	8 (0.8)	0.68 (0.29-1.61)	.38

*PY*, Person-year; *HR*, hazard ratio; *CI*, confidence interval; *aHR*, adjusted hazard ratio; *COPD*, chronic obstructive pulmonary disease.

**Figure 2:**
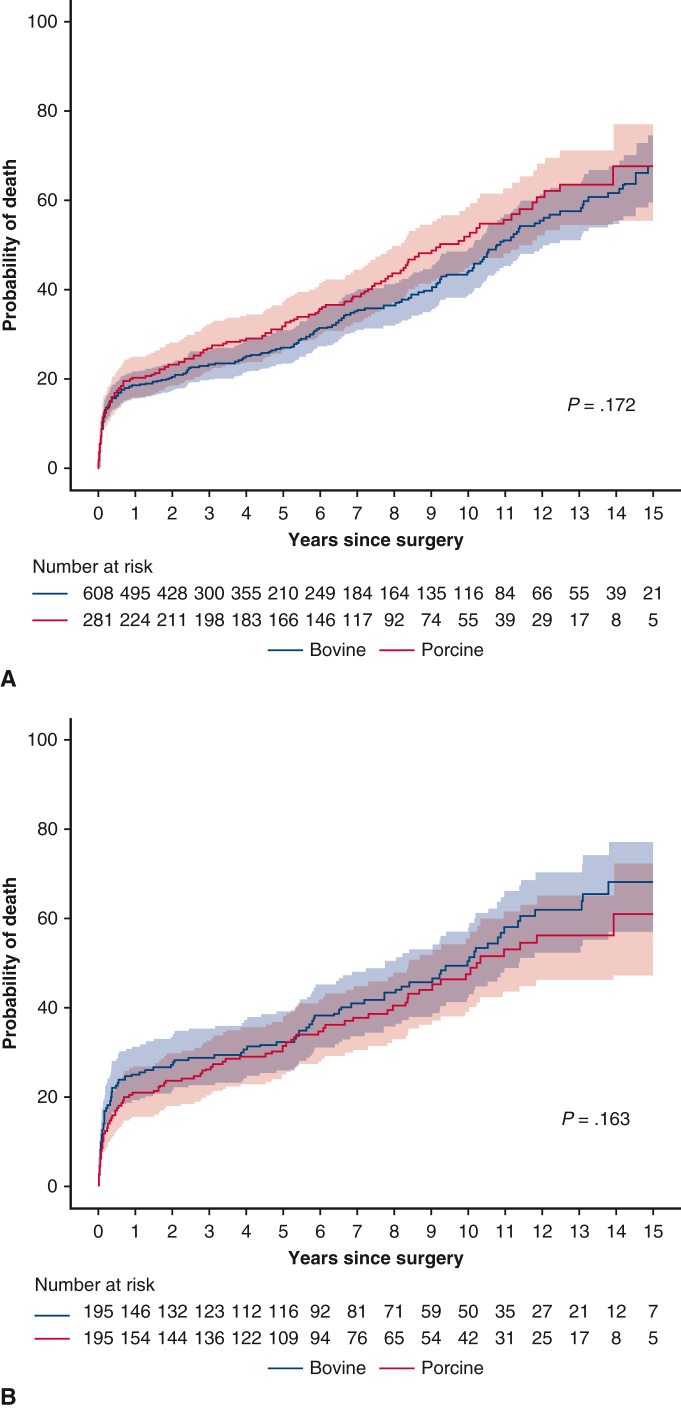
Cumulative incidence of death during follow-up in the overall (A) and propensity score–matched (B) cohorts. The *shaded* area represents the 95% confidence interval.

In the unadjusted analysis, cardiovascular death rates were 4.8%/PY in the bovine group and 4.9%/PY in the porcine group, and all-cause death rates were 7.5%/PY and 8.0%/PY, respectively. No statistically significant differences were found between the bioprosthetic valve groups in terms of cardiovascular mortality (hazard ratio [HR], 1.16; 95% confidence interval [CI], 0.94-1.43) or all-cause mortality (HR, 1.10; 95% CI, 0.84-1.45) (Figure 2, *A*). In competing risk analysis, the risk of endocarditis was higher in the porcine group (HR, 2.82; 95% CI, 1.07-7.45), whereas no significant between-group differences were observed in other valve-related events (ie, reoperation, thromboembolism, and hemorrhage).

**Figure 3:**
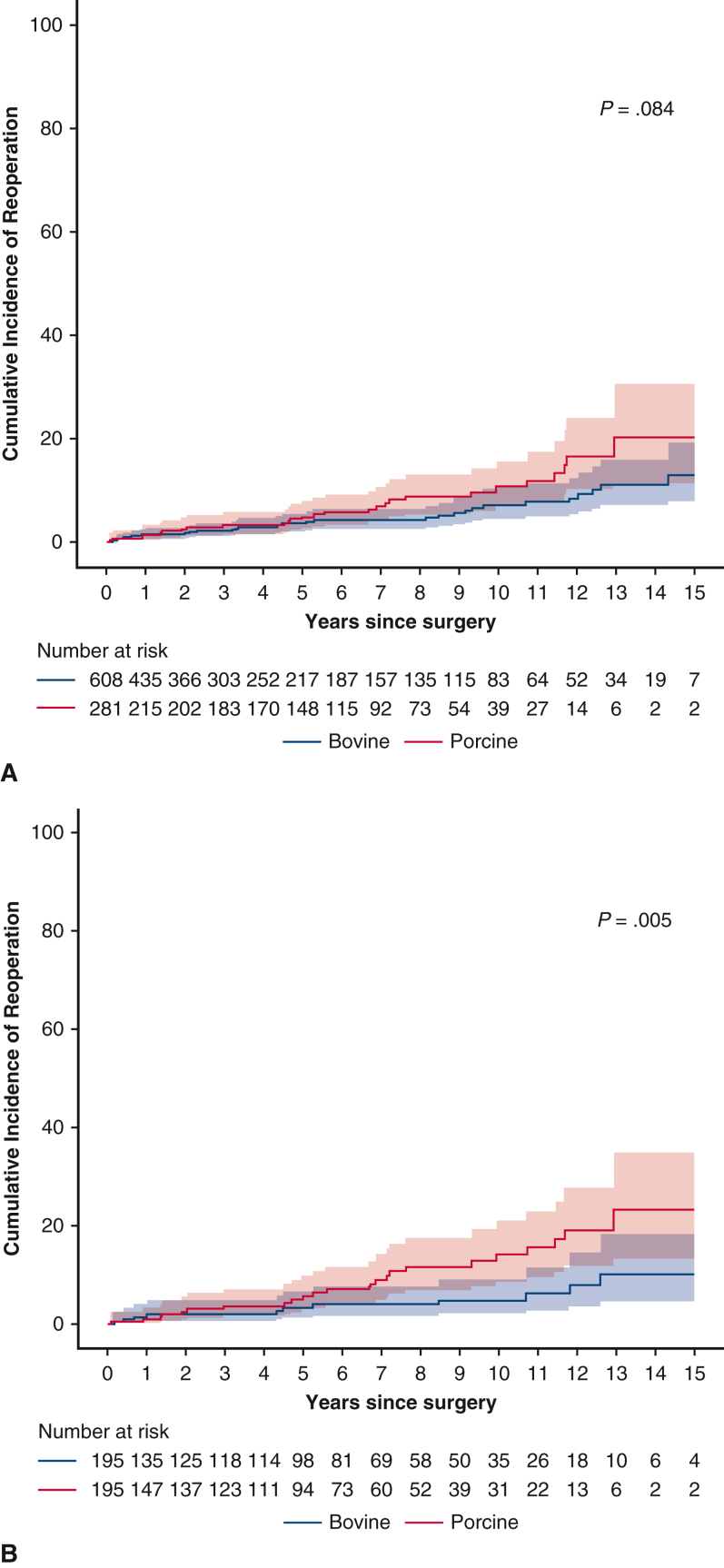
Cumulative incidence of reoperation in the overall (A) and propensity score–matched (B) cohorts. The *shaded* area represents the 95% confidence interval.

After PS matching, cardiovascular mortality (adjusted HR [aHR], 0.74; 95% CI, 0.50-1.07) and all-cause mortality (aHR, 0.87; 95% CI, 0.65-1.17) did not differ significantly between the bovine and porcine groups (Figure 2, *B*). Furthermore, no significant differences were seen in other valve-related events, including endocarditis (HR, 2.31; 95% CI, 0.59-9.10), thromboembolism (aHR, 0.88; 95% CI, 0.40-1.93), and hemorrhage (aHR, 1.07; 95% CI, 0.76-1.53). However, patients with porcine valves had a higher risk of reoperation (aHR, 2.08; 95% CI, 1.10-3.94) in competing risk analysis (Figure 3).

Table E4 summarizes reoperation rates according to valve type. In the entire patient cohort, there were 59 reoperations during the follow-up period, including 32 in the bovine group and 27 in the porcine group. The average delay between index surgery and reoperation was 5.69 ± 4.85 years for the bovine group and 5.86 ± 3.89 years for the porcine group. DVR was the most common reoperation performed in both groups, followed by MVR and AVR. There was 1 death in each group within 30 days after reoperation. Additionally, there was no significant between-group difference in 30-day mortality after reoperation (odds ratio, 1.19; 95% CI, 0.07-20.01).

### Subgroup Analysis

An adjusted subgroup analysis using inverse probability of treatment weighting was performed to investigate cardiovascular mortality and reoperation rates according to valve type across different clinical risk subgroups (Table 4). There were no significant differences in cardiovascular mortality rates based on the choice of valve prosthesis observed across all subgroups; however, patients who received a porcine valve and did not have diabetes mellitus had a higher risk of reoperation (*P* for interaction = .027), as did those who received a porcine valve and had a Charlson comorbidity index (CCI) <2 (*P* for interaction = .043).

**Table 4:** IPTW-adjusted subgroup analysis for the primary endpoints

Endpoint	Bovine group (N = 608)	Porcine group (N = 281)	HR	95% CI	*P* value	*P* value for interaction
Cardiovascular mortality						
Age						
<70 y	66/254	22/116	0.683	0.375-1.246	.21	.44
≥70 y	103/354	46/165	0.918	0.590-1.427	.70	
Sex						
Male	79/241	33/110	0.839	0.527-1.337	.41	.91
Female	90/367	34/171	0.805	0.480-1.350	.41	
Diabetes mellitus						
No	135/478	45/219	0.690	0.467-1.020	.063	.08
Yes	34/130	23/62	1.352	0.718-2.547	.35	
Stroke, SE						
No	139/511	52/231	0.764	0.521-1.120	.17	.40
Yes	30/97	16/50	1.126	0.492-2.578	.78	
Congestive heart failure						
No	79/316	42/155	1.073	0.653-1.761	.78	.10
Yes	90/292	26/126	0.604	0.376-0.971	.037	
Atrial fibrillation						
No	115/362	42/165	0.779	0.512-1.184	.24	.66
Yes	54/246	25/116	0.926	0.491-1.749	.81	
CCI						
<2	59/240	26/116	0.915	0.520-1.612	.76	.65
≥2	110/368	41/165	0.777	0.501-1.205	.26	
Reoperation						
Age						
<70 y	22/254	22/116	2.293	1.08-4.88	.031	.79
≥70 y	10/354	13/165	2.736	0.96-7.78	.059	
Sex						
Male	18/241	12/110	1.391	0.55-3.53	.49	.12
Female	14/367	23/171	3.740	1.61-8.70	.002	
Diabetes mellitus						
No	25/478	33/219	3.133	1.64-5.99	.001	.027
Yes	7/130	2/62	0.539	0.13-2.23	.39	
Stroke, SE						
No	29/511	33/231	2.503	1.32-4.75	.005	.56
Yes	3/97	2/50	1.483	0.30-7.60	.64	
Congestive heart failure						
No	17/316	20/155	2.595	1.24-5.44	.012	.78
Yes	15/292	15/126	2.169	0.80-5.96	.13	
Atrial fibrillation						
No	23/362	22/165	2.145	1.03-4.47	.041	.61
Yes	9/246	14/116	3.047	1.00-9.30	.050	
CCI						
<2	15/240	26/116	4.094	1.97-8.50	<.001	.043
≥2	17/368	9/165	1.131	0.42-3.07	.81	

*IPTW*, Inverse probability of treatment weighting; *HR*, hazard ratio; *CI*, confidence interval; *SE*, systemic embolization; *CCI*, Charlson comorbidity index.

## Discussion

This nationwide cohort study demonstrates that the choice of bioprosthetic valve type was not correlated with an increased risk of cardiovascular mortality, all-cause mortality, or valve-related events, including endocarditis, thromboembolism, and hemorrhage events after DVR. However, the use of porcine valves during DVR was associated with a higher risk of reoperation compared to the use of bovine valves.

Previous studies have reported better hemodynamic profiles of bovine valves compared to porcine valves; however, this advantage did not translate into a survival benefit or improved survival or clinical outcomes.^8^^,^^9^ In our previous population-based study focusing on the mitral position, we found no significant differences in cardiovascular mortality or valve-related events between the two valve types, which is consistent with other investigations.^10^ However, in the aortic position, findings regarding survival from previous national population-based studies have been mixed. An England-based national registry study did not detect a significant difference in survival between the 2 valve types, while a Swedish population-based study reported improved survival with porcine valves compared to bovine valves.^11^^,^^12^ Conversely, studies on reoperation generally have reported a higher rate of structural valve deterioration with porcine prostheses.^12^^,^^13^ Our Korean population-based study on AVR found no notable difference in cardiovascular mortality between the 2 valve types; however, consistent with previous findings, the use of porcine prostheses was significantly associated with an elevated risk of reoperation compared to the use of bovine prostheses.^6^

In the present study on DVR, we found no significant difference in cardiovascular mortality between the 2 bioprosthesis valve groups; however, the rate of reoperation was higher in the porcine valve group. Considering the results of previous studies on each valve type, the absence of a significant difference in cardiovascular mortality aligns with expectations. The findings regarding reoperation suggest that the aortic position may have a crucial role in the outcomes after DVR, potentially exerting a greater influence than the mitral position.

In previous studies, type 2 diabetes mellitus and metabolic syndrome have been associated with a rapid progression of mean gradient and may contribute to structural valve degeneration following AVR.^14^^,^^15^ In our previous investigation focusing on AVR, we observed a survival advantage associated with the use of bovine valves over porcine valves among patients without diabetes mellitus. However, in the current study, we found no disparity in cardiovascular mortality between the 2 valve types irrespective of the presence or absence of diabetes. This discrepancy with the findings of the AVR study may stem from several factors, including the performance of subgroup analysis, specifically on cardiovascular mortality in the current study. Moreover, inherent limitations of retrospective analysis and potential differences in hemodynamics between patients undergoing AVR and DVR, similar to those observed in MVR patients, could contribute to these divergent results. Intriguingly, unlike cardiovascular mortality, the reoperation rate was higher among patients without diabetes in the porcine valve group. This suggests a potential association between the divergent clinical outcomes of the 2 valve types based on the presence or absence of diabetes. Further investigation is warranted to elucidate this relationship more comprehensively.

Furthermore, in patients with lower CCI scores, the use of porcine valves was associated with a higher rate of reoperation. Given that lower CCI scores often correlate with better overall health and fewer comorbidities, leading to a longer life expectancy, the subsequent need for additional surgeries potentially could exacerbate the observed difference in reoperation rates between porcine and bovine valves.

### Limitations and Strengths

This study investigated a nationwide population using data from the NHIS, a comprehensive insurance program covering nearly the entire South Korean population. A notable strength of this dataset lies in its ability to provide long-term follow-up data, even in cases of patient relocation or changes in healthcare providers. Moreover, the NHID includes a wide range of health screening information, such as baseline blood tests, body measurements, and self-reported questionnaires.

The study has some limitations that should be acknowledged. First, because of its retrospective nature, selection bias might have influenced the study results. However, we endeavored to mitigate this bias through propensity score matching and the use of falsification endpoints. Second, owing to the nature of code-based national data, detailed information on prosthetic valves could not be obtained; in particular, researchers were not granted access to detailed valve product names and sizes. Consequently, all pericardial and all porcine valves were grouped together for analysis. As a result, we were unable to assess the impact of individual valve characteristics, such as annular versus supra-annular design, effective orifice area, and preservation techniques, as well as the potential effects of valves that were withdrawn early from the market. However, Trifecta and Mitroflow valves were used to only a limited extent in the Korean market, and thus their impact on study outcomes is expected to be minimal. Consequently, establishing a direct causal relationship between the use of specific valve types and outcomes was challenging.

## Conclusions

Our nationwide retrospective cohort study on DVR shows that the selection of bioprosthetic valve type was not associated with an increased risk of cardiovascular mortality. However, the use of porcine prostheses was associated with a higher risk of reoperation.

## Conflict of Interest Statement

The authors reported no conflicts of interest.

The *Journal* policy requires editors and reviewers to disclose conflicts of interest and to decline handling or reviewing manuscripts for which they may have a conflict of interest. The editors and reviewers of this article have no conflicts of interest.

## Figures and Tables

**Table E1:** Details of bioprosthetic valves

Prosthesis name	NHIS claim code
Porcine valves	
Hancock II valve	G2001003
Mosaic tissue valve	G2001103
SJM Epic valve	G2001121
Epic Supra valve	G2001221
SJM BIOCOR porcine valve	G2001021
Toronto SPV valve	G2001007
Bovine pericardial valves	
Soprano pericardial heart valve	G2001034
Carpentier Edwards Perimount Magna TFX valve	G2001102
Carpentier Edwards Perimount valve	G2001002
Pericarbon More pericardial heart valve	G2001134
Avalus bioprosthesis	G2001203
Trifecta valve	G2001321
Mitroflow aortic pericardial heart valve, Crown PRT aortic pericardial heart valve	G2001234
Rapid deployment/sutureless valve	
Intuity Elite valve system	G2301002
Perceval S	G2301034

*NHIS*, National Health Insurance Service.

**Table E2:** Definition of baseline comorbidities and operative covariates

Comorbidities	ICD-10 codes	NHIS claim code	Number of diagnoses/additional definition
Hypertension	I10-I13, I15	-	Admission or outpatient clinic ≥3
Diabetes mellitus	E10-E14	-	Admission or outpatient clinic ≥3
Dyslipidemia	E78	-	Admission or outpatient clinic ≥3
Atrial fibrillation	I48		Admission or outpatient clinic ≥1
Chronic kidney disease	N18	-	Admission or outpatient clinic ≥2
Dialysis	-	O701x-O708x	Admission or outpatient clinic ≥1
Stroke/TIA/SE	I63, I64, I67.8, I67.9, G45, I74		Admission or outpatient clinic ≥2
Ischemic heart disease	I20-I25	-	Admission or outpatient clinic ≥2
Myocardial infarction	I21-I23	-	Admission or outpatient clinic ≥2
Previous PCI	-	M6551, M6552, M6561-M6564, M6571, M6572	Admission or outpatient clinic ≥1
Congestive heart failure	I50, I42, I11.0, I13.0, I13.2	-	Admission or outpatient clinic ≥2
Anemia	D50-D64	-	Admission or outpatient clinic ≥2
COPD	J44	-	Admission or outpatient clinic ≥2
Asthma	J45	-	Admission or outpatient clinic ≥2
Peripheral vascular disease	I70	-	Admission or outpatient clinic ≥2
Previous cardiac surgery	-	O1660, O1671, O1672, O1680, O1701-O1705, O1710, O1711, O1721-O1723, O1730, O1740, O1750, O1760, O1770, O1781-O1783, O1791, O1800, O1810, O1821-O1826,O1830, O1840, O1851, O1852, O1861, O1873-O1875, O1878, O1879, O0881-O0883, O1940, O1950, O1960, O1970, O1981, O1982, O2001, O2004, O2006, O2007, O1640, O1641, OA640, OA641, O1648, OA648, O1649, OA649, O1647, OA647, O2031-O2033	Admission ≥1
Previous cancer	C00-C97	-	Admission or outpatient clinic ≥1, and cancer registration codes (V027, V193, V194)
Concomitant procedure			
Surgical ablation		O2006	
TV repair		O1781	
CABG		O1641, O1642, O1647, OA641, OA642, OA647	

*ICD-10*, International Classification of Diseases, Tenth Revision; *NHIS*, National Health Insurance Service; *TIA*, transient ischemic attack; *SE*, status epilepticus; *PCI*, percutaneous coronary intervention; *COPD*, chronic obstructive pulmonary disorder; *TV*, tricuspid valve; *CABG*, coronary artery bypass grafting.

**Table E3:** Definition of clinical outcomes

Comorbidities	ICD-10 codes	NHIS claim code	Additional definition
Outcomes			
Endocarditis	T82.7, T826 I33, I38, I39.0, I39.1, I39.4, I39.8		Until the end of follow-upOperated valve endocarditis
Reoperation	-	O1795	Until the end of follow-upMitral valve reoperation
Thromboembolism	I63,64,74	HE101, HE201, HE501, HE102, HE502, HE135, HE235, HE535, HE136, HE236, HE301, E302, HF101, HF201, HF102, HF202, HF103, HF203, HF104, F105, HF35, HF106, HF306, HF107, HA441, HA45, HA461, HA471, HA851, HA601, HA60, HA603, HA604, HA605	Hospitalization, brain imaging (CT or MRI code), primary or subsidiary diagnosis
Hemorrhage	-		
Hemorrhagic stroke	I60-I62	HE101,HE201,HE501,HE102,HE502,HE135,HE235,HE535,HE136,HE236,HE301,E302,HF101,HF201,HF102,HF202,HF103,HF203,HF104,F105,HF35,HF106,HF306,HF107,HA441,HA45,HA461,HA471,HA851,HA601,HA60,HA603,HA604,HA605	Hospitalization, brain imaging (CT or MRI) with primary or subsidiary diagnosis
Major bleeding	I85.0, K22.1, K22.8, K25.0, K25.2, K25.4, K25.6, K26.0, K26.2, K26.4, K26.6, K27.0, K27.2, K27.4, K27.6, K28.0, K28.2, K28.4, K28.6, K29.0, K31.8, K55.2, K57.0, K57.1-K57.5, K57.8, K57.9, K62.5, K66.1, K92.0, K92.1, K92.2, D62, H05.2, H35.6, H43.1, J94.2, M25.0, R04		Hospitalization with primary or subsidiary diagnosis, gastrointestinal bleeding or hemorrhagic event occurring at unclassified sites (eg, extracranial, intraocular, intra-articular, hemothorax) requiring hospitalization
Falsification endpoint			
COPD	J44	-	Admission to ER or hospitalization with primary or subsidiary diagnosis
Pneumonia	J10.0, J11.0, J12-J18, A48.1	-	Admission to ER or hospitalization with primary or subsidiary diagnosis
Lower extremity fracture	S32.1-S32.5, S32.7, S32.8, S32.82, S32.88, S32.89, S72, S82	-	Admission to ER or hospitalization with primary or subsidiary diagnosis

*ICD-10*, International Classification of Diseases, Tenth Revision; *NHIS*, National Health Insurance Service; *CT*, computed tomography; *MRI*, magnetic resonance imaging; *COPD*, chronic obstructive pulmonary disorder; *ER*, emergency room.

**Table E4:** Reoperations according to valve type

Reoperation valve	Bovine (N = 608)	Porcine (N = 281)	Total
MVR	8	3	11
AVR	1	2	3
DVR	23	22	45

*MVR*, Mitral valve replacement; *AVR*, aortic valve replacement; *DVR*, double-valve replacement.
